# Incidence of postoperative acute kidney injury in dogs without pre-existing renal disease

**DOI:** 10.3389/fvets.2025.1483101

**Published:** 2025-03-26

**Authors:** Lorena Muñoz-Blanco, Verónica Salazar

**Affiliations:** ^1^Department of Veterinary Medicine, School of Biomedical and Health Sciences, Universidad Europea de Madrid, Villaviciosa de Odon, Spain; ^2^Department of Anesthesia and Analgesia, Veterinary Teaching Hospital, Universidad Alfonso X El Sabio, Madrid, Spain

**Keywords:** AKI, dogs, postoperative, surgery, anesthesia, creatinine, acquired AKI

## Abstract

Acute kidney injury (AKI) is defined as a sudden reduction in renal function, characterized by a rapid increase in serum creatinine (sCr) ≥ 0.3 mg/dL within 48 h with or without azotemia (sCr ≥ 1.7 mg/dL) and/or oliguria (urinary output <1 mL/kg/h for more than 6 h). Acute kidney injury is associated with increased mortality, prolonged hospitalization, and higher costs in both human and veterinary medicine. This study aimed to determine the incidence of postoperative AKI in dogs without pre-existing renal disease. A total of 170 dogs, admitted for elective surgery (ASA I-II) at a single university center, were included. The sCr levels were measured at the following times: procedure day (before anesthesia), 24 h, 48 h, and 7 days post-surgery (0 h, 24 h, 48 h, and 7d). Potential risk factors for AKI including patient characteristics (age, sex, pathologies, treatments), anesthetic protocol (drugs, type and rate of fluid therapy, procedure duration) and intraoperative complications were analyzed. Postoperative AKI was identified in 5 dogs (2.9, 95% CI: 1.3–6.7%) based on a sCr increase ≥0.3 mg/dL within 48 h post-surgery. A decrease in sCr (Mean: 0.87 SD = 0.2) was observed at 48 h (Mean: 0.84 SD = 0.24) (*p* < 0.001), returning to baseline by day 7 (Mean:0.89 SD = 0.22) (*p* = 0.127). Only a relationship between surgery duration and the probability of developing postoperative AKI was found (*p* = 0.037). Further studies are warranted to identify risk factors for AKI in dogs undergoing GA and improve its prevention optimizing postoperative management and prognosis.

## 1 Introduction

Acute kidney injury (AKI) is defined as a sudden reduction in normal renal function, characterized by an absolute increase in serum creatinine (sCr) ≥ 0.3 mg/dL (≥ 26.4 μmoL/L) within 48 h after general anesthesia and/or a reduction in urine output (<1 mL/kg/h for more than 6 h). The AKI may be reversible depending on the degree and duration of renal damage and can occur without apparent loss of renal function (1, 2).

The lack of evidence regarding the incidence of postoperative AKI in dogs without pre-existing renal disease highlights the need for further investigation. Understanding the occurrence of AKI in the broader population is essential, as postoperative AKI can have significant impacts on patient outcomes. In human medicine, AKI is recognized as a common complication during the postoperative period, various mechanisms have been proposed to explain this association. These include hemodynamic changes (hypotension, hypovolemia, and decreased renal perfusion during surgery), as well as nephrotoxic effects of certain anesthetic agents. Additionally, inflammation, oxidative stress, and microvascular injury induced by surgery and anesthesia can contribute to renal damage (3, 4). However, there is limited understanding of how these mechanisms might apply to dogs undergoing anesthesia, especially those without pre-existing renal disease.

The AKI is associated with an increased morbidity and mortality (5–7), as well as prolonged hospitalization and/or the need for more intensive treatments, resulting in higher costs (8). The incidence of postoperative AKI in human medicine varies between 1 and 36% depending on the type of surgery and the definition used for AKI. In non-cardiac surgery, incidence ranges from 0.8 to 10%, while in patients undergoing cardiac surgery, this incidence can reach 36% (9–11).

Acute kidney injury is recognized in human medicine as a multifaceted systemic disease with significant impacts on distant organ function, including heart, lungs, brain, liver, and immune system. Evidence suggests that AKI contributes directly to remote organ injury through various mechanisms. Endotoxin translocation occurs following intestinal barrier disruption associated with AKI, leading to hepatic inflammation and systemic cytokine release. Cerebral dysfunction, including uremic encephalopathy, results from activation of neuroinflammatory cascade that increases vascular permeability of the blood–brain barrier. In the cardiac system, AKI is linked to cardiorenal syndrome, (simultaneous heart and kidney failure), driven by factors such as fluid overload and uremia-related reductions in myocardial contractility. Pulmonary effects include activation of inflammatory cascade that increase vascular permeability and neutrophil infiltration, causing pulmonary edema. Furthermore, AKI impairs immune function through oxidative stress and reduced cytokine clearance, increasing infection risk. These systemic effects highlight the complex and far-reaching consequences of AKI (3, 4).

In veterinary medicine, data on postoperative AKI, particularly in dogs without pre-existing renal disease, is scarce. Most studies have focused on the overall incidence of hospital-acquired AKI, encompassing various etiologies including anesthesia. These studies report an incidence of 9 to 14.5% of hospitalized patients (5, 12). Notably, hospital-acquired AKI is associated with a high mortality rate, ranging from 45 to 62%, even with advanced treatments such as hemodialysis (8, 13). This underscores the potential severity of AKI, warranting a deeper investigation into its postoperative incidence, particularly in low-risk population as those without diagnosed renal disease.

In healthy dogs, an increase in urinary *γ*-glutamyltransferasa (GGT) activity has been observed within 24 h after surgery, although the presence of renal dysfunction or failure could not be demonstrated (14). More recently, a postoperative AKI (defined as creatinine >1.60 mg/dL according to IRIS guidelines) (15) incidence ranging from 0.09% (4/4480) to 0.17% (6/3542) has been reported over two consecutive years in healthy dogs undergoing elective surgery. These dogs were considered healthy based on their medical history and physical examination (16).

The aim of this study was to prospectively report the incidence of postoperative AKI in a sample of dogs patients without pre-existing renal disease undergoing elective surgical procedures, classified from ASA I - II, at university referral animal hospital.

As a secondary objective, we also aimed to understand the normal progression of serum creatinine levels in these patients following general anesthesia.

The hypothesis proposed in this study is that postoperative AKI can occur in dogs without pre-existing diagnosable renal disease following general anesthesia.

## 2 Materials and methods

### 2.1 Animals

A prospective observational longitudinal study was conducted, including dogs scheduled for general anesthesia at university referral animal hospital between March 2019 and March 2022. A signed informed consent was obtained from all owners of dogs enrolled. The study was approved by the Ethics Committee of Alfonso X El Sabio University.

Inclusion criteria: dogs undergoing elective procedure under general anesthesia, classified ASA I–II, and for which informed consent was obtained from the owner.

Exclusion criteria included: Patients presenting any alteration during pre-anesthetic evaluation in terms of serum creatinine (sCr) values >1.4 mg/dL (reference value internal laboratory), urinalysis, or urine protein-to-creatinine ratio (UPC) >0.5 indicating renal function abnormalities. Also, those previously diagnosed with chronic kidney disease or scheduled for urinary system surgery, due to the potential test results alteration caused by surgery.

Additionally, aggressive dogs were excluded due to the difficulty and stress involved in obtaining samples for the study.

### 2.2 Procedures

#### 2.2.1 Sample collection

All patients underwent a complete physical examination, complete blood count, blood biochemistry, and urinalysis collected by cystocentesis before anesthetic premedication. All dogs´ previous pathologies and current medications were recorded. Blood samples were collected at the following times: day of the procedure (before anesthesia premedication), and at 24 h, 48 h, and 7 days after anesthesia (0 h, 24 h, 48 h, and 7d). sCr was analyzed on 1 mL of whole blood collected in Lithium Heparin (Tapval^TM^ Tube, Aquisel^®^, Spain) and measured using an automated spectrophotometer (Cobas Integra^®^ 400 plus, Roche Farma, S.A., Spain).

Dogs were classified as having AKI if serum creatinine increased by ≥0.3 mg/dL at 24 and/or 48 h post-procedure relative to baseline creatinine. Patients were classified as non-AKI if this increase was not observed.

#### 2.2.2 Anesthetic procedure

There were four anesthetists involved in the study; however, no significant differences in the management of complications are expected, as all followed the directives of the department head and worked in a highly standardized and consistent manner according to 2020 AAHA Anesthesia and Monitoring Guidelines for Dogs and Cats (17).

Owners were advised to ensure that their dogs fasted for at least 6 h for solids and 2 h for liquids. The anesthetic protocol was tailored to each patient according to their clinical characteristics and the scheduled procedure. The type of fluid therapy was also recorded, including the type of fluids administered, such as balanced isotonic solutions (Isofundin or Ringer Lactate) or unbalanced solutions (Normal Saline), and the rate of administration for each patient. Intravenous fluids were administered at a rate of 5 mL/kg/h. However, in dogs with known cardiac disease, the rate was reduced to 2–3 mL/kg/h depending on the type and degree of pathology. Patients with intraoperative hypotension who were deemed to benefit from additional fluids received a bolus of fluids at 5 or 10 mL/kg over 20 min. This was indicated in cases such as intraoperative bleeding or suspected mild dehydration (not clinically relevant before anesthesia, but which could be exacerbating intraoperative hypotension), as recommended by the AAHA 2013 guidelines (18). The bolus was recorded as an increase in the administration rate to up to 6–7 mL/kg/h, depending on the total amount of fluids administered (18). The total amount of fluids received by each patient was also recorded. Hospitalization time was based on procedure performed (inpatient surgery). In such cases, they received postoperative fluid therapy, had free access to water, and a regular feeding schedule. Patients who did not require postoperative hospitalization (outpatient surgery) were discharged the same day and did not receive postoperative fluid therapy. Outpatient dogs returned to the hospital at 24 h, 48 h, and 7d postoperatively for blood sample collection.

During general anesthesia, all vital signs of the patient were monitored and recorded: heart rate (HR) and rhythm using electrocardiography, respiratory rate (RR), oscillometric non-invasive blood pressure (Vet30, SunTech Medical, United States), expired CO_2_ (EtCO_2_) (Vamos plus capnograph, Dräger, Drägerwerk AG & Co., KGaA, Germany), oxygen saturation (SpO_2_), inspired fraction of oxygen (FiO_2_), and esophageal temperature (Temp) (Vista 120 multiparameter monitor, Dräger, Drägerwerk AG & Co., KGaA, Germany) every 5 min. Any intervention performed was also recorded.

Intraoperative complications included: hypotension (median arterial pressure < 60 mmHg for at least 5 min), bradychardia (HR < 40 bpm for at least 5 min), hypoventilation (EtCO_2_ > 50 mmHg for at least 15 min), hypoxemia (SpO_2_ < 95% for at least 15 min), or hypothermia (temperature < 36.5°C for at least 15 min during the procedure). Hypotension was treated at discretion of the anesthetist with the administration of isotonic crystalloid bolus and/or synthetic colloids (5 mL/kg of ISOHES) and/or constat rate infusion (CRI) of positive inotropes (dobutamine CRI) and/or vasopressors (norepinephrine CRI), or atropine administration if bradycardia was considered the cause of hypotension. In case of hypoventilation positive pressure ventilation (PPV) was initiated or modified to maintain EtCO_2_ < 50 mmHg. In case of hypoxemia, the FiO_2_ was incremented to 1 if necessary, and PPV with PEEP was initiated or adjusted. If hypoxemia was not corrected with these steps, alveolar recruitment maneuvers were performed.

### 2.3 Statistical analysis

A descriptive analysis of all variables was performed, and the Shapiro–Wilk test was used to assess normal distribution. Continuous data were presented as mean ± standard deviation (SD), if they followed a normal distribution, or as median and interquartile range (median [Q1, Q3]) if they did not. Categorical data were described as frequencies.

A descriptive analysis of baseline sCr and its change (absolute and relative) at 24 h, 48 h, and 7d was performed for all enrolled dogs, and for animals with and without AKI separately. The incidence of AKI was calculated from the study sample, and a 95% confidence interval (CI) was estimated to provide the range within which the true incidence likely falls in the broader population. A paired Student’s *t*-test was conducted to analyze whether there were significant differences in sCr levels between each time point and baseline, for the entire population as well as for those in the AKI and non-AKI groups, separately.

Potential factors associated with the incidence of AKI were: age, gender, weight, body condition score (BCS, points 0–9), pre-existing medical conditions (yes or no), ongoing treatments prior to anesthesia (treatments, y/n), procedure duration, anesthetic duration, administered drugs (NSAIDS, intravenous contrast, colloid fluids, type and rate of fluid therapy and total fluids received), complication (y/n), hypotension (y/n) and hypotension duration and postoperative fluid therapy (y/n). And were analyzed using the chi-square test or Fischer’s exact test for categorical variables and the Mann Whitney *U*-test for continuous variables. Univariate (and if viable, also multivariate) logistic regression models were considered to assess the magnitude of association (relative risk, RR, and its 95%CI) for the variables showing significant association. For continuous variables with non-parametric behavior, we determined the empirical optimal cutoff point using the Receiver Operating Characteristic (ROC) curve (Liu Method) to dichotomize these variables and calculate the relative risk of AKI incidence.

*p* ≤ 0.05 was considered statistically significant. Calculations were performed using a statistical software package (STATA).

## 3 Results

A total of 232 dogs were enrolled in the study, of these 62 did not meet inclusion criteria, one presented with azotemia (0.41%), 61 presented an UPC ≥ 0.5 (26.3%), resulting in 170 patients finally being included in the study. Of these, 52% (89/170) were male (of which 39% (39/89) were neutered) and 48% (81/170) were female (of which 44% (44/81) were neutered). The main breeds were: Mixed dogs 40 (23%), Labrador 14 (8%), German Shepherd 9 (6%), Beagle 8 (5%), and Boxer, French Bulldog, Yorkshire, Schnauzer, and Maltese Bichon, each with 7 (4%) patients. The remaining 32% were distributed among 30 different breeds, with between 1 and 6 (<3.5%) patients per breed. The mean age was 7 ± 4 years. Twenty-three percent of the patients presented at least one pre-existing condition (cardiovascular, endocrine, infectious, or other conditions) and 49.4% of the total patients were on treatment (Table 1).

**Table 1:** Demographic and clinical variables of patients finally included in the study.

Variables	Total patients (170)
Age, years (mean, SD)	7 (± 4)
Weight, kg (mean, SD)	20 (± 13.2)
Gender (n and %)	
Male	89 (52%)
Female	81 (48%)
Pre-existing medical conditions (n and %)	
None	130 (76.47%)
Cardiovascular conditions *Heart murmur* *MMVD B1* *MMVD B2* *Subaortic stenosis*	14 (8.24%) 1 8 4 1
Endocrine disease *Hypothyroidism* *Addison* *Cushing* *Diabetes Mellitus*	6 (3.5%) 3 1 1 1
Infectious diseases *Leishmaniasis*	11 (6.5%) 11
Others *Seizures* *Meningitis* *Discospondylitis* *Esophageal dilation* *Bening prostatic hyperplasia* *Hypercalcemia*	9 (5.3%) 4 1 1 1 1 1
Treatments (n and %)	
None	83 (48.8%)
NSAIDs	18 (10.6%)
Antibiotics	5 (2.9%)
Others/combinations**	64 (37.65%)

**Other/combinations: NSAIDS + antibiotics, prednisone, levothyroxine, trilostane, insulin, allopurinol, osaterone acetate, imepitoin, potassium bromide, phenobarbital, levetiracetam, tranexamic acid, ursodeoxycholic acid, omeprazole, metoclopramide, maropitant, ranitidine, gabapentin, tramadol, methadone, acetaminophen, ketamine, monoclonal antibody, trazodone, pimobendan, benazepril, spironolactone (just one dog in non-AKI group) and masitinib.

A significant difference was observed between baseline sCr and sCr at 24 (*p* < 0.001) and 48 h (*p* < 0.001) analyzing the entire sample. No difference was found between baseline creatinine and sCr at 7 days (*p* = 0.127) (Table 2).

**Table 2:** Variation in serum creatinine concentration relative to baseline at 24 h, 48 h, and 7 days post-surgery, for the entire population and subgroups with or without AKI.

	Entire populati*o*n (*n* = 170)	Patients without AKI (*n* = 165)	Patients with AKI (*n* = 5)
Baseline			
Creatinine (mg/dL)	0.87 ± 0.21 (170)	0.87 ± 0.21	0.83 ± 0.24
Urea (mg/dL)	34.08 ± 10.40		
24 h post-surgery			
Creatinine (mg/dL)	0.79 ± 0.24 (169)	0.78 ± 0.23	1.04 ± 0.17
Absolute change (mg/dL)*	−0.08 ± 0.16	−0.09 ± 0.15	0.21 ± 0.27
Relative change (%)*	−8.25 ± 20.27	−9.36 ± 18.52	28.28 ± 39.45
*p*-value**	**<0.001**	**<0.001**	0.150
48 h post-surgery			
Creatinine (mg/dL)	0.81 ± 0.23 (164)	0.80 ± 0.22	1.23 ± 0.37
Absolute change (mg/dL)*	−0.06 ± 0.16	−0.07 ± 0.14	0.40 ± 0.14
Relative change (%)*	−5.68 ± 20.00	−7.41 ± 17.63	49.07 ± 12.82
*p*-value**	**<0.001**	**<0.001**	**0.003**
7 days post-surgery			
Creatinine (mg/dL)	0.90 ± 0.22 (142)	0.90 ± 0.22	0.93 ± 0.29
Absolute change (mg/dL)*	0.02 ± 0.15	0.02 ± 0.15	0.03 ± 0.20
Relative change (%)*	3.50 ± 17.98	3.50 ± 17.98	4.21 ± 24.15
*p*-value**	0.127	0.139	0.766

*With respect to baseline serum creatinine value.

**Student’s *t*-test for paired samples (Creatinine at 24 h, 48 h or 7 days compared to baseline).

Bold numbers indicate statistically significant *p*-values (*p* < 0.05).

**Figure 1:**
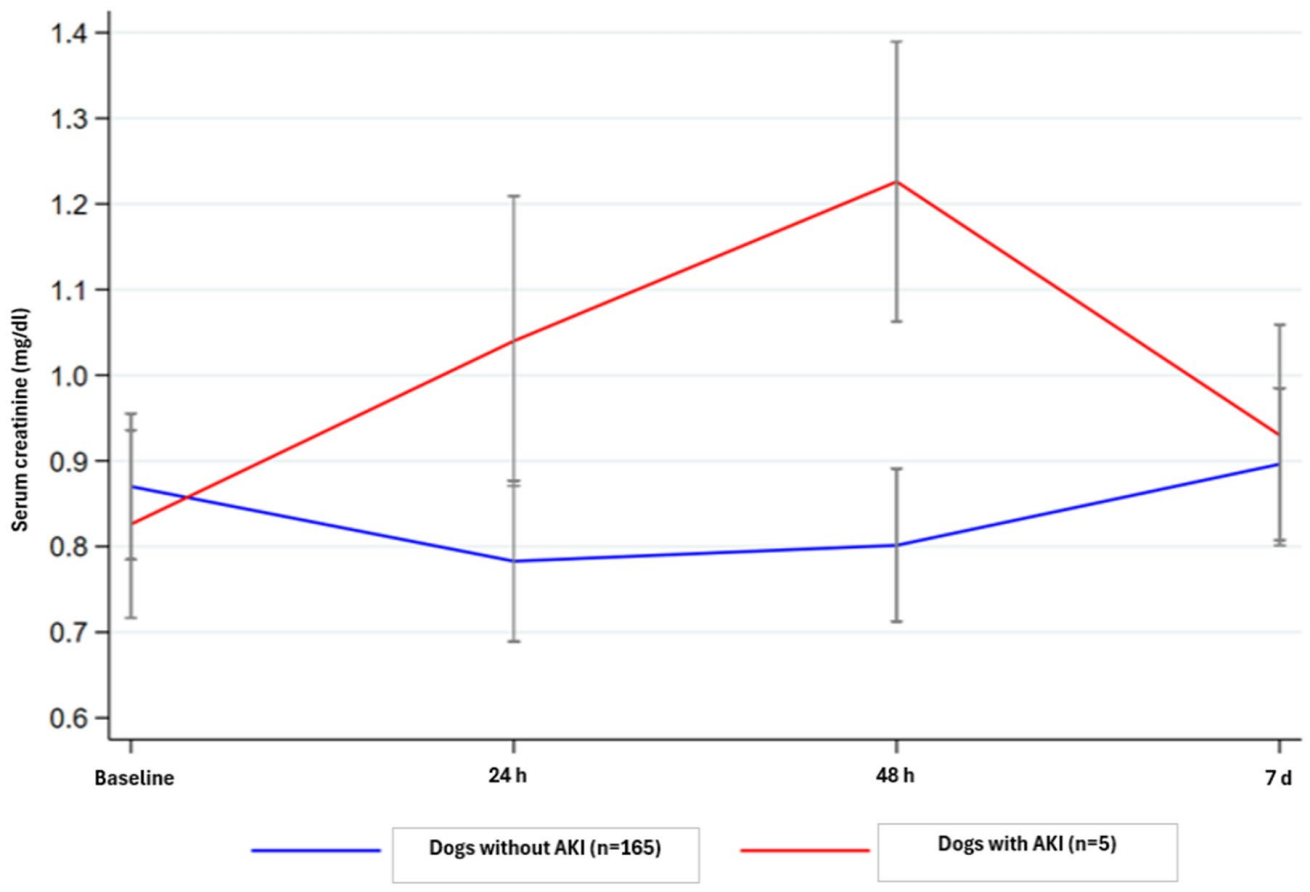
Evolution of serum creatinine (Mean ± SEM) over the 7 days following surgery in animals that developed or did not develop AKI (increase ≥0.3 mg/dL) within 48 h post-surgery).

Analyzing the patients with or without AKI separately, a significant difference was also observed in both groups in the serum creatinine values at 24- and 48-h post-procedure, returning to near baseline levels by day 7 (Table 2). In the group without AKI (165/170), the trend shows a decrease in serum creatinine values after general anesthesia, whereas in the AKI group (5/170), there is an increase of nearly 50% in the mean value at 48 h (Figure 1).

Five patients (2.9%; 95% CI: 1.3–6.7%) experienced an increase of ≥0.3 mg/dL in serum creatinine compared to baseline within 48 h post-procedure, which was considered postoperative AKI. Of these, three developed AKI within the first 24 h. Two of these patients maintained an increase in sCr ≥0.3 mg/dL compared to their baseline values over the first 48 h, while the third had a creatinine difference of 0.23 mg/dL at 48 h compared to their baseline value. Regarding the patients in whom AKI was detected at 48 h, one showed an increase in creatinine of 0.21 mg/dL compared to their baseline value at 24 h, while the other had a decrease of 0.21 mg/dL compared to baseline. It is noteworthy that the latter was the only patient among the five who developed AKI that remained hospitalized during the first 24 h and, therefore, received fluid therapy during this period. Since this was an observational study and the results were only assessed after obtaining the final sample from each patient, no treatment was administered to these patients.

The dogs that developed AKI were classified as ASA I, except for one, which was classified as ASA II due to the presence of stage 2 of myxomatous mitral valve disease (MMVD-B2). This dog was admitted for routine periodontal treatment involving tooth extractions. Of the remaining four patients, two underwent trauma surgery, one underwent a total ear canal ablation (TECA), and the last patient underwent laparoscopic ovariectomy. The latter was the youngest, aged 1 year, while the others were between 8 and 11 years old.

When assessing the association of the studied variables with the incidence of AKI, procedure duration was found to be significantly shorter in animals with AKI (35 [20, 39] minutes) compared to those without AKI (58 [37, 89] minutes; *p* = 0.037). Animals with surgeries lasting less than 40 min (empirical cutoff point, area under the ROC curve = 0.76; sensitivity = 0.75; specificity =0.80) showed a significantly higher incidence of AKI (8.16% vs. 0.83%; RR = 9.88; 95% CI: 1.13, 86.12).

No significant associations were found between the development of AKI and patient age (*p* = 0.418), gender (*p* = 0.728), body condition (*p* = 0.620), presence of pre-existing conditions (*p* = 0.828), or any treatment (*p* = 0.631) (Table 3).

**Table 3:** Comparison of clinical characteristics, surgical and anesthetic times, drugs and fluids administration, and intraoperative complications between dogs with and without AKI.

	Patients without AKI (*n* = 165)	Patients with AKI (*n* = 5)	*p*-value
Demographic and clinical characteristics			
Gender, male (n and %)^‡^	86 (52.1%)	3 (60.0%)	0.728
Age, years (median [Q1, Q3])*	7 [4, 10]	10 [8, 11]	0.418
Weight, kg (median [Q1, Q3])*	17.6 [9.0, 31.1]	8.6 [7.6, 23.8]	0.294
BCS, points 0–9 (median [Q1, Q3])*	5 [5, 6]	6 [5, 6]	0.620
Pre-existing conditions (yes, n and %)^‡^	39 (24.2%)	1 (20.0%)	0.828
Treatments (yes, n and %)^‡^	81 (49.1%)	3 (60.0%)	0.631
Procedure and anesthetic times			
Procedure duration, min (median [Q1, Q3])*	58 [37, 89]	35 [20, 39]	**0.037**
Anesthetic duration, min (median [Q1, Q3])*	113 [66, 149]	90 [80, 105]	0.249
Sort of procedures (n and %)			
Traumatologic	34 (20.6%)	2 (40%)	–
Dentistry	31 (18.8%)	1 (20%)	–
Neurologic	25 (15.6%)	0 (0%)	–
Soft tissue	47 (28.5%)	1 (20%)	–
Laparoscopy/thoracoscopy	21 (12.7%)	1 (2%)	–
Imaging Diagnostics	7 (4.2%)	0 (0%)	–
Drugs^#^			
NSAIDs (yes, n and %)^‡^	103 (62.4%)	3 (60.0%)	0.912
Colloids (yes, n and %)^‡^	7 (4.2%)	0 (0%)	0.638
Intravenous contrast (yes, n and %)^‡^	9 (5.5%)	0 (0%)	0.592
Fluid therapy			
Total volume administered during anesthesia, ml (median [Q1, Q3])*	141.8 [53.3, 306.5]	79.9 [64.5, 177.3]	0.407
Fluid rate administered during anesthesia, ml/kg/h (median [Q1, Q3])*	5 [5, 5]	5 [5, 5]	0.332
Postoperative Fluid therapy (yes, n and %)^‡^	60 (36.4%)	1 (20.0%)	0.452
Intraoperative complications			
At least one complication (yes, n and %)^‡^	103 (62.4%)	2 (40.0%)	0.309
Hypotension (yes, n and %)^‡^	56 (33.9%)	0 (0%)	0.112
Hypotension duration, min (mean ± SD)*	5.3 ± 9.9	0 ± 0	0.235

*Mann Whitney U test to compare between dogs with and without AKI of quantitative variables.

^‡Chi-square or Fisher’s exact test to compare between dogs with and without AKI of quantitative variables.^

Bold numbers indicate statistically significant *p*-values (*p* < 0.05).

^#^Other drugs in the anesthetic protocol: dexmedetomidine (83.5%), acepromazine (17.6%), methadone (92.9%), butorphanol (3.5%), buprenorphine (3.5%), fentanyl (62.3%), remifentanil (3.53%), midazolam (87%), ketamine (35.8%), cephazolin (82.3%), amoxicillin (2.35%), methylprednisolone (3.5%), atipamezole (2.35%), isoflurane (90%), sevoflurane (9.45%), propofol (76.4%), alfaxalone (10%), thiopental (9.4%), etomidate (1.76%).

AKI: acute kidney injury. Q1: 1st Quartile (25th percentile); Q3: 3rd Quartile (75th percentile). Points on body condition: 0–9, where 5 represents optimal body condition.

Sixty-one-point seven percent (105/170) of the patients experienced at least one complication, with no relationship observed between the occurrence of one or more complications in the same patient and the development of postoperative AKI (*p* = 0.309). Thirty-three-point nine percent (56/170) of the patients experienced hypotension, with a mean duration time of 5.3 ± 9.9 min, without a relationship with the incidence of AKI (*p* = 0.112), nor with the duration time of hypotension (*p* = 0.235) (Table 3).

No significant relationship was observed between the drugs used in the anesthetic protocols and the likelihood of developing AKI. Similarly, there was no significant association with the administration or non-administration of NSAIDS (*p* = 0.912), colloids (*p* = 0.638), or intravenous contrast (*p* = 0.592) (Table 3).

## 4 Discussion

In this unicentric prospective study, conducted at university referral animal hospital, with dogs with normal preoperative renal function undergoing an anesthetic procedure, an incidence of 2.9% (5/170, 95% CI: 1.3–6.7%) of AKI was found. According to IRIS guidelines, AKI is defined as an increase in sCr ≥ 0.3 mg/dL within 48 h. The incidence of AKI in human medicine in patients undergoing non-cardiac surgery ranges from 0.8 to 10% according to different studies (10, 11, 19–21). In veterinary medicine, most studies focus on the general incidence of hospital-acquired AKI and analyze all possible etiologies together: infectious, toxicological, ischemic/inflammatory injuries (including postoperative AKI), or unknown. According to different studies, this incidence varies from 9 to 14.6% (5, 12).

Specifically in canine species, one study has evaluated the incidence of postoperative AKI. In this retrospective study, with a total population of 3,542 dogs anesthetized at a single center, 0.17% (*n* = 6) were diagnosed with postoperative AKI (according to the IRIS guidelines) when they presented to the emergency department 4 days (2–14 days) after undergoing scheduled general anesthesia (16). In our case, the incidence is much higher, which can be explained due to the retrospective nature of their study, in which only patients who presented to the emergency department with azotemia within 2 weeks after non-emergency surgery were counted as AKI cases among all patients anesthetized at the center during the year analyzed. Given the prospective nature of our study, we obtained the same parameters for all patients. This allowed for the diagnosis of acquired AKI in all patients analyzed. The absence of clinical symptoms, even in cases of postoperative AKI, would therefore exclude patients, thus underestimating the real incidence of the disease (16).

In another study an incidence of 4.3% of postoperative AKI in dogs underwent desexing surgery was observed (22). This result is more in line with ours, indeed the study design is very similar, as both studies are prospective, based on IRIS guidelines and blood samples are obtain at the same time points.

In our study, we analyzed the variation in creatinine levels during the first 48 h after general anesthesia, which is considered a standard test for the diagnosis of AKI (15). Serum creatinine serves as a surrogate marker for glomerular filtration rate and is classified as a functional biomarker, similar to symmetric dimethylarginine (SDMA). Both are considered insensitive indicators in the early stages of AKI, as significant reductions in glomerular filtration rate are required before serum creatinine levels rise. Furthermore, AKI can occur without a loss of renal function, limiting the value of these biomarkers in such cases. Numerous emerging renal biomarkers (urinary biomarkers of renal tubular cells) aim to improve the early diagnosis of AKI, detecting kidney injury before functional loss occurs. It is reasonable to expect that some of these new biomarkers will become clinically available soon (23). However, the validation of their specificity and diagnostic value remains a work in progress.

In our study, a decrease in sCr was observed in the first 48 h after general anesthesia when analyzing the entire population as a whole. In another study, with healthy patients undergoing general anesthesia, no variation in creatinine levels was observed (14). This difference could be attributed to the fact that patients in that study did not receive fluid therapy during anesthesia, unlike our case. It has been shown that fluid therapy increases glomerular filtration rate but does not proportionally increase urine production; the amount of fluids received is greater than urinary output, resulting in a positive fluid balance in the patient, which translates into increased creatinine clearance (24).

On the other hand, in humans, a 10% decrease in serum creatinine levels has been observed during the 5 days following major surgery. The authors relate this decline to the reduction in body mass index and dietary changes during postoperative hospitalization (11). In dogs, it has been observed that fasting for 10–26 h does not cause variation in certain biochemical parameters, including creatinine (25). Most of our patients were fasted for less than 24 h, including the preoperative fasting period, and underwent outpatient surgeries with a hospitalization duration of less than 12 h, without requiring strict rest. Therefore, it is unlikely that the decrease in serum creatinine was solely due to muscle loss or alterations in dietary habits. Instead, a plausible alternative hypothesis is that the decrease in sCr is caused from dilution due to positive fluid balance, as previously discussed.

In different studies, an increase in sCr of 0.2 mg/dL has been associated with increased morbidity and mortality (10). This could be related to the fact that in most patients after general anesthesia, as in our study, serum creatinine decreases; therefore, even minimal increases, lower than 0.3 mg/dL, could have clinical relevance.

Surgery time is considered a risk factor for AKI in human medicine (11, 21, 26). In our study, we found an inverse relationship between longer surgery times and the likelihood of developing postoperative AKI (*p* = 0.037). This association might be explained by the larger fluid volumes typically administered during extended surgeries, which could enhance creatinine clearance. However, our findings did not identify a significant link between the total volume of fluids administered and AKI development (*p* = 0.407).

Additionally, patients undergoing longer procedures were more likely to require hospitalization than those with shorter surgeries. Hospitalized patients (inpatients: 61/170) received fluid therapy during the postoperative period, unlike those discharged earlier (outpatients: 109/170). Among these groups, only 1 inpatient developed AKI, compared to 4 outpatients. However, no significant differences were observed in fluid volume, infusion rates, or postoperative fluid therapy when comparing the AKI and non-AKI groups.

In human patients, factors such as age (neonates or geriatrics), sex, obesity, corticosteroid treatment, diabetes, and cardiovascular diseases have been associated with the development of postoperative AKI (11, 27). However, no such relationship has been found in veterinary medicine (7, 16). In our case, we also could not find a relationship between patient factors (neither pre-existing medical conditions nor treatments), as well as with the drugs used in the anesthetic protocol, the type of surgery, or the occurrence of complications during the intraoperative period. Regarding hypotension, none of the patients who developed AKI in our study experienced this condition, which may explain the lack of association between hypotension and AKI in this case. Although experimental studies in dogs have shown that severe hypotension (MAP <40 mmHg for 1 h) can lead to renal injury biomarkers (28), clinical studies in veterinary medicine have not been able to demonstrate a clear link between different risk factors or intraoperative complications and the development of AKI (7, 29). Furthermore, although intraoperative hypotension has been clearly linked to AKI in human medicine, such as MAP <55 mmHg for over 10 min or MAP <60 mmHg for 11–20 min (30), findings in veterinary medicine are less consistent, potentially due to differences in renal physiology, compensatory mechanisms, or study populations.

This study has several limitations that should be acknowledged. The inclusion criteria did not account for age differences, as both neonatal and geriatric dogs were included, which may introduce variability in creatinine values due to age-related physiological differences. Additionally, no exclusion criteria were applied regarding breed or BCS. However, this decision was made because the primary focus was on the variation in creatinine levels rather than the absolute values, thereby limiting the potential impact of breed-related differences on creatinine measurements.

Furthermore, hydration and volemic status were not specifically evaluated or corrected, as the study included clinically stable dogs with normal hematocrit and total protein values. These factors should be addressed in future research or noted as potential limitations in interpreting the findings of this study.

Due to its unicentric nature, this study may face a lack of representativeness (selection bias) and the potential introduction of uncontrolled factors (confounding bias) that could influence the incidence of AKI, as it was conducted in routine clinical practice. We observed an incidence of postoperative AKI of 2.9% (95% CI: 1.3–6.7%), which means that only 5 of the 170 animals included in the study were affected. Such a low number of animals with AKI greatly hinders the identification of factors associated with the disease. This highlights the need for large-scale (multicentric) studies that include a substantial number of dogs with and without AKI to ensure sufficient statistical power. Such studies are necessary not only to clarify and gather more information about the association between AKI and the duration of surgery suggested by our results, but also to explore other potential associations. These include the volume and rate of fluid therapy, as well as factors such as age, treatments and comorbidities like hypertension, congestive heart failure, pulmonary disease, diabetes mellitus, peripheral vascular disease, ascites, and obesity, as reported in various studies in human medicine (9, 20, 26, 31). Furthermore, future studies might consider that, while early diagnosis and treatment initiation could improve patient prognosis, patients in the early stages of AKI often go unnoticed due to a lack of clinical signs, and thus continuous assessment of sCr might be necessary.

This study represents an initial approach to the incidence of postoperative AKI in dogs. Additionally, the data shows that the normal progression of sCr after a procedure under general anesthesia is to decrease during the first 48 h, returning to baseline values at day 7.

## Data Availability

The raw data supporting the conclusions of this article will be made available by the authors, without undue reservation.
